# Impact of virtual avatar appearance realism on perceptual interaction experience: a network meta-analysis

**DOI:** 10.3389/fpsyg.2025.1624975

**Published:** 2025-12-03

**Authors:** Zhiyu Tao, Yanyan Liu, Junsheng Qiu, Shengwei Li

**Affiliations:** Graduate School of Design, Kyushu University, Fukuoka, Japan

**Keywords:** virtual avatars, appearance realism, perceptual experience, attractiveness, trustworthiness, eeriness, network meta-analysis

## Abstract

**Objective:**

As virtual avatars become increasingly embedded in social, educational, and commercial platforms, the virtual avatars appearance realism (VAAR) has emerged as a critical factor influencing user perceptual interaction experience. This study explores the impact of VAAR on users’ perceptual interaction experiences.

**Method:**

We retrieved all relevant publications from the past decade (April 2015 to April 2025) across four major databases: Web of Science, Elsevier Science Direct, Springer Link, and Google Scholar. The analysis focused on three user perceptual interaction experience-related indicators: attractiveness, trustworthiness, and eeriness. A network meta-analysis (NMA) was conducted using Stata software, and the relative ranking of VAAR was determined based on the surface under the cumulative ranking curve.

**Results:**

A total of 13 studies with 2,343 participants were included in the analysis. According to the results of the NMA, high VAAR were perceived as more attractive and more trustworthy than those with medium or low VAAR. In contrast, medium VAAR elicited the strongest feelings of eeriness.

**Conclusion:**

This study highlights the subjective impact of VAAR on user interaction experience. These findings provide meaningful guidance for the future development and design of virtual avatars.

**Systematic review registration:**

The network meta-analysis (NMA) was preregistered on the Open Science Framework (OSF) and is publicly available at https://doi.org/10.17605/OSF.IO/5TRG6.

## 1 Introduction

In recent years, the widespread use of head-mounted displays has significantly changed how people engage in virtual social interactions, enabling more immersive participation (Smith and Neff, 2018). To deliver more realistic visual effects and provide experiences in virtual environments that closely mirror those in the real world, enhancing the virtual avatars appearance realism (VAAR) of virtual avatars has become a prominent trend. Tools such as Character Creator have substantially improved the efficiency of avatar generation and are now capable of achieving near-photorealistic fidelity (Gao et al., 2025). At the same time, users’ perceptions of VAAR have grown increasingly complex and multidimensional, directly influencing both their overall experience and the quality of their interactions with virtual avatars. Therefore, systematically investigating the impact of avatar realism on user experience holds considerable significance.

As the basis for users’ aesthetic judgments and first impressions of virtual avatars, visual attractiveness plays a crucial role in perceptual interaction and can rapidly shape users’ willingness to further engage with the avatars (Bacev-Giles and Haji, 2017; Tuch et al., 2012). The evaluation of attractiveness is largely determined by perceptual expectations, such as balanced body proportions and the naturalness of facial features. When these expectations are met, virtual avatars are more likely to be perceived as visually appealing (Dubosc et al., 2021; Fraser et al., 2024; Gao et al., 2025; Kim et al., 2023). Therefore, higher levels of VAAR are generally associated with stronger visual attractiveness (Oyekoya and Baffour, 2025; Zibrek et al., 2019).

In addition, in many virtual environments designed to simulate reality, virtual avatars have increasingly taken on functions that closely resemble social roles in the real world. In such interactions, trustworthiness is a critical factor in determining whether users are willing to accept guidance or establish sustained engagement (Wills et al., 2018). Its importance is particularly evident in high-trust domains such as education and healthcare. For instance, in virtual classrooms, the perceived trustworthiness of virtual instructors directly shapes students’ learning motivation and knowledge acquisition (Inal and Cagiltay, 2006; Oyekoya and Baffour, 2025; Segaran et al., 2021). Similarly, in virtual medical consultations, trustworthiness determines whether patients are willing to follow medical advice, thereby influencing treatment adherence (Dai and MacDorman, 2021). Prior research indicates that high VAAR and strong alignment with their expected social roles are associated with higher perceived trustworthiness (Canales et al., 2024).

However, increasing virtual realism does not always lead to positive outcomes. In entertainment and game-oriented contexts, users often favor avatars with lower VAAR, such as stylish or cartoon-like designs. Evidence indicates that such avatars can reduce social anxiety and facilitate social interaction (Ma and Pan, 2022; Segaran et al., 2021). On the other hand, when virtual avatars exhibit high appearance realism but lack natural coordination in detail, they may trigger the uncanny valley effect (Dubosc et al., 2023; MacDorman et al., 2009; Shin et al., 2019). This effect has been shown to evoke feelings of eeriness, discomfort, or even aversion, thereby weakening users’ emotional connection with virtual avatars (MacDorman et al., 2009; Mori et al., 2012; Seymour et al., 2021; Shin et al., 2019). As we noted earlier, users’ perceptions of avatar realism have become increasingly complex and multidimensional; therefore, merely enhancing visual realism does not necessarily guarantee a positive user experience.

Previous studies have also explored additional subjective dimensions to further examine how the VAAR influences user experience. For instance, some studies have introduced social presence (Dubosc et al., 2021; Volante et al., 2016) and preference (Pan et al., 2024; Shih et al., 2023) as indicators. Social presence emphasizes whether users experience a sense of co-presence and interaction with avatars, and preference reflects users’ overall evaluations across different avatar designs. However, these dimensions operate at a relatively macro level and are easily influenced by factors such as voice, movement, or task context, which makes it difficult to isolate the specific contribution of VAAR to perception and interaction.

Therefore, this study focuses on three dimensions—visual attractiveness, trustworthiness, and eeriness—and employs a network meta-analysis (NMA) to systematically synthesize high-quality empirical findings. Specifically, VAAR is categorized into three levels: (1) high realism avatars (HRA)—characterized by highly detailed skin textures, human-like proportions, and human-like anatomical structures; (2) medium realism avatars (MRA)—featuring basic human proportions but with more stylized or blurred characteristics; and (3) low realism avatars (LRA)—marked by exaggerated facial features and simplified contours, commonly presented in cartoon, sketch, or comic styles.

The primary objective of this study is to examine the relationship between different levels of VAAR and user perceptual interaction experience by synthesizing findings from previous research. The findings are intended to support the avatar design and provide theoretical guidance for improving user interaction with virtual avatars.

## 2 Methods

This NMA was preregistered on the Open Science Framework and is publicly available at https://doi.org/10.17605/OSF.IO/5TRG6.

### 2.1 Search strategy

A comprehensive literature search was conducted across four databases: Web of Science, Elsevier Science Direct, Springer Link, and Google Scholar. The search covered studies published over the past 10 years (April 2015 to April 2025). Boolean operators “OR” and “AND” were used to combine search terms. Specifically, the keywords included: “Virtual Human,” “Digital Human,” “Virtual Character,” “Virtual Avatars,” “Appearance Realism,” “Visual Realism,” “Photorealism,” and “Facial Realism.” The detailed search strategy is provided in Supplementary material 1(a).

### 2.2 Inclusion and exclusion criteria

Studies were included if they met the following conditions: (1) written in English; (2) published as peer-reviewed journal articles or conference proceedings; (3) designed to compare at least two levels of VAAR; and (4) reported at least one user experience outcome—specifically related to attractiveness, trustworthiness, or eeriness—measured using an assessment scale.

Studies were excluded if they met any of the following criteria: (1) reviews or qualitative articles; (2) duplicate publications; (3) failure to report relevant outcome variables; (4) insufficient data for extraction; or (5) lack of a clear description of the VAAR.

### 2.3 Data extraction

Data extraction was performed using a pre-specified data extraction form by two independent reviewers. Any discrepancies between the reviewers were resolved through discussion or by consulting a third reviewer. The extracted information included: first author, year of publication, country or region, sample size, number of female participants, mean age, type of VAAR, and outcome measures related to attractiveness, trustworthiness, and eeriness.

To ensure consistency and reproducibility in the classification of VAAR, this study designed a four-dimensional rating scale. The scale systematically evaluates the VAAR across four aspects: skin and texture details, facial proportions and anatomical accuracy, body proportions and skeletal structure, and degree of stylization. The total score was then calculated and mapped into three categories: 5–11 points: LRA, 12–18 points: MRA, and 19–25 points: HRA. The specific evaluation details are provided in Supplementary material 1(b).

For each of the outcome measures, the mean values, standard deviations, and corresponding sample sizes were retrieved and entered a standardized spreadsheet. In cases where relevant data were missing, study authors were contacted for clarification. When data were available only in graphical form, the software Plot Digitizer (Slashdot Media, San Diego, CA, USA) was used to extract mean values and standard deviations from the figures.

### 2.4 Quality assessment

The methodological quality of the included studies was independently assessed by two reviewers using the guidelines proposed by Kitchenham and Charters (2007). A total of 10 questions were used to evaluate each study design, implementation, analysis, and conclusions within the context of meta-analysis. Each item was scored as “1” for “Yes” and “0” for “No,” resulting in a total quality score ranging from 0 to 10. Only studies with a score greater than or equal to 8 were included in the NMA. The methodological quality assessment items and corresponding results for the included studies are provided in Supplementary material 1(c).

### 2.5 Statistical analysis

The NMA was conducted using Stata software (version 16). The outcomes in this study were treated as continuous variables, and the effect sizes were estimated using standardized mean differences along with 95% confidence intervals. Statistical significance was considered when the *p*-value was below 0.05.

Global inconsistency and node-splitting analyses were conducted to evaluate the incoherence of the network. A consistency model was applied only when both tests indicated no significant inconsistency (*p* > 0.05). If any inconsistency was detected within the network, a sensitivity analysis was carried out to locate its source, and the corresponding study was removed from the network. Funnel plots were used to assess publication bias within each network. Surface under the cumulative ranking curve (SUCRA) values were used to rank different levels of VAAR.

## 3 Results

### 3.1 Literature search

**Figure 1:**
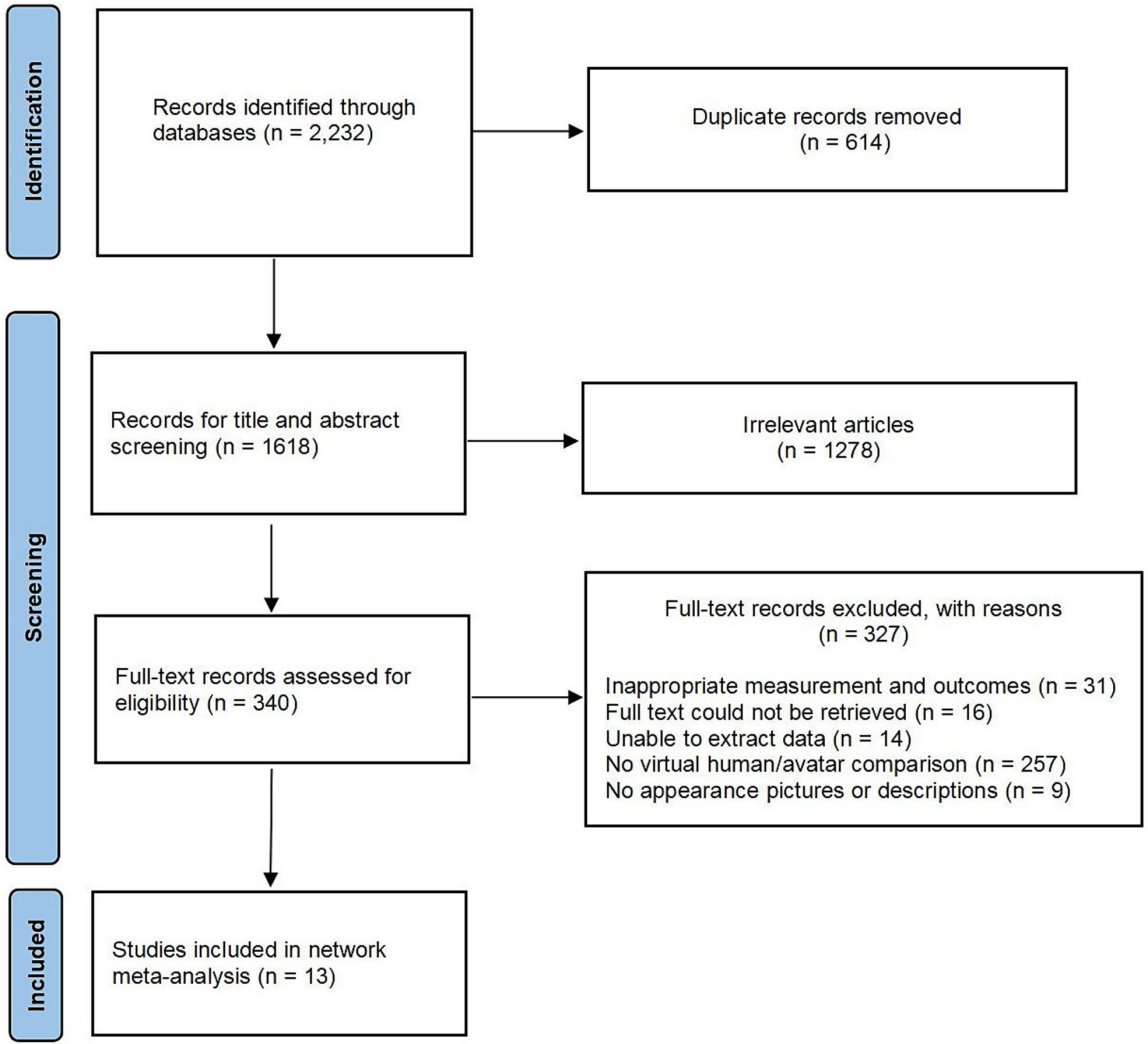
Literature screening process.

A total of 2,232 relevant articles were initially identified across all databases. After screening titles and abstracts, followed by a full-text review, 13 studies met the inclusion criteria and were included in this NMA. The detailed selection process is illustrated in Figure 1.

### 3.2 Basic characteristics of included studies

A total of 2,343 participants were included in the NMA. The proportion of female participants was relatively low, and two studies included only male participants (Gorisse et al., 2018; Kokkinara and McDonnell, 2015). The studies originated from various countries, including China (Gao et al., 2025), Netherlands (Amadou et al., 2023), Australia (Fraser et al., 2024), France (Dubosc et al., 2023; Gorisse et al., 2018), Ireland (Kokkinara and McDonnell, 2015; Zibrek et al., 2018), Germany (Mal et al., 2024; Stein and Ohler, 2018; Straßmann and Krämer, 2018), and the United States (Canales et al., 2024; Cornelius et al., 2023; Seymour et al., 2019). The basic characteristics of each study are summarized in Supplementary material 1(d).

### 3.3 Consistency analysis results

Inconsistency tests were performed for the 13 included studies. The global inconsistency results were no significant for attractiveness (*p* = 0.319), trustworthiness (*p* = 0.947), and eeriness (*p* = 0.326). Local inconsistency was further evaluated using the node-splitting method, which also indicated no significant inconsistency (*p* > 0.05). Therefore, a consistency model was applied for the subsequent analysis.

### 3.4 Results of network meta-analysis

**Figure 2:**
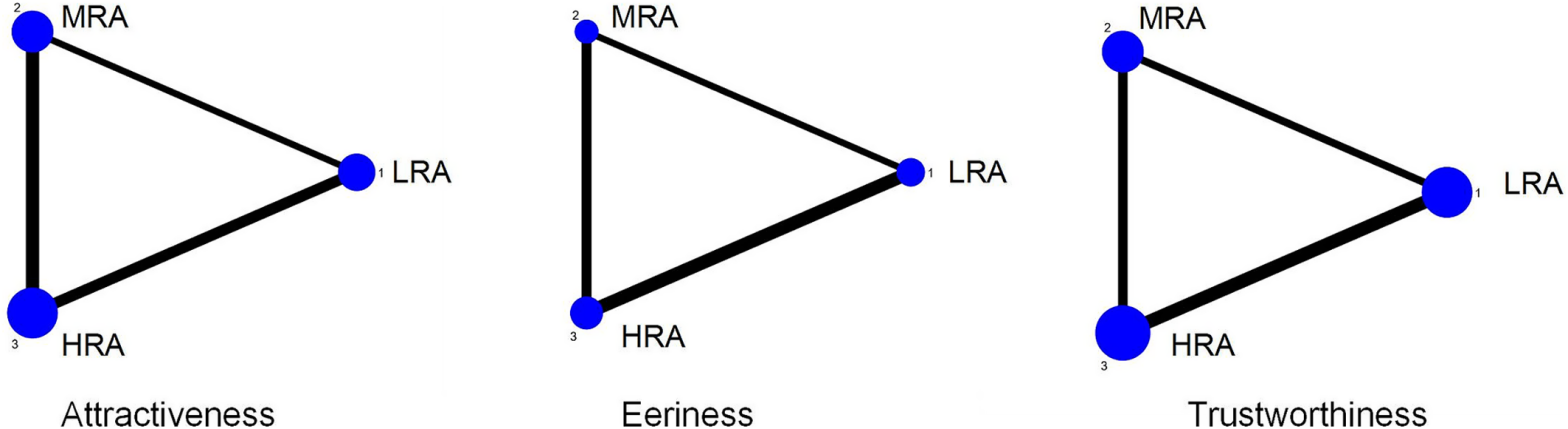
Network geometry comparing high realism avatars (HRA), medium realism avatars (MRA), and low realism avatars (LRA).

Evidence networks were constructed separately for the three user experience outcomes: attractiveness, eeriness, and trustworthiness. In the network diagrams, each dot represents a VAAR condition, and the size of the dot reflects the total sample size associated with that condition. Lines between dots indicate direct comparisons between two VAAR conditions, with the thickness of each line corresponding to the number of studies contributing to that comparison. The network plots are shown in Figure 2.

### 3.5 Comparative analysis of visual attractiveness

**Figure 3:**
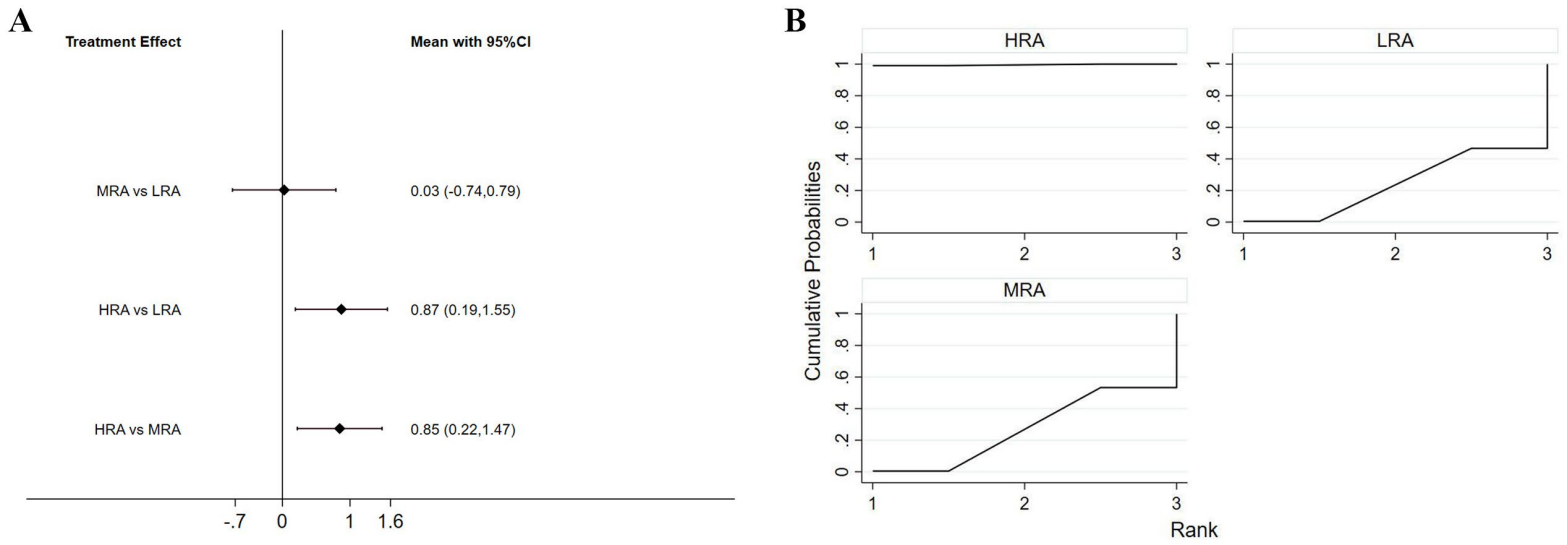
**(A)** Interval diagram based on visual attractiveness. **(B)** Surface under the cumulative ranking curve based on visual attractiveness.

Eight studies (61.5%) assessed visual attractiveness. The NMA results indicated that the HRA received significantly higher ratings than both the MRA (*p* = 0.008) and the LRA (*p* = 0.012). No significant difference was observed between the MRA and LRA groups (Figure 3A).

In the SUCRA ranking of different levels of VAAR for visual attractiveness, the HRA had the highest probability of being the most attractive (99.5%), followed by MRA (26.9%) and LRA (23.6%) (Figure 3B).

### 3.6 Comparative analysis of trustworthiness

**Figure 4:**
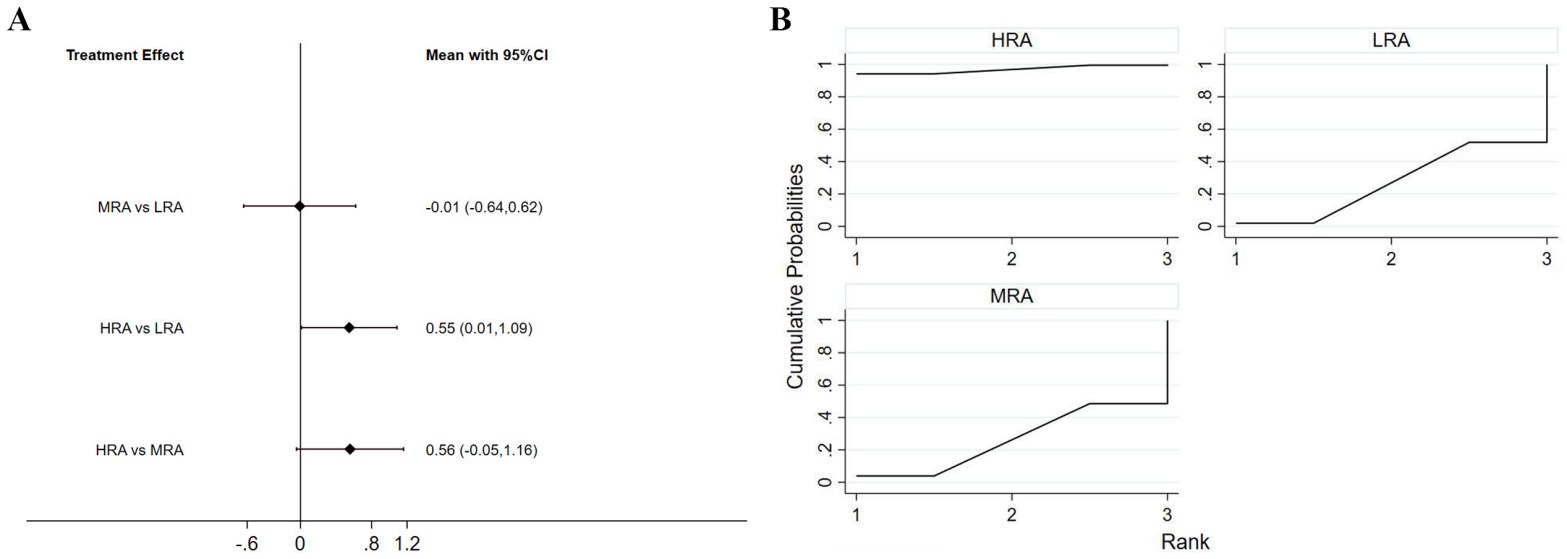
**(A)** Interval diagram based on trustworthiness. **(B)** Surface under the cumulative ranking curve based on trustworthiness.

Eight studies (61.5%) included comparisons of trustworthiness. The NMA results indicated that the HRA received significantly higher ratings than the LRA (*p* = 0.046). No significant differences were observed between the MRA group and either the HRA or LRA groups (Figure 4A).

In the SUCRA ranking of different levels of VAAR for trustworthiness, the HRA had the highest probability of being the most trustworthy (96.2%), followed by MRA (29.9%) and LRA (23.9%) (Figure 4B).

### 3.7 Comparative analysis of eeriness

**Figure 5:**
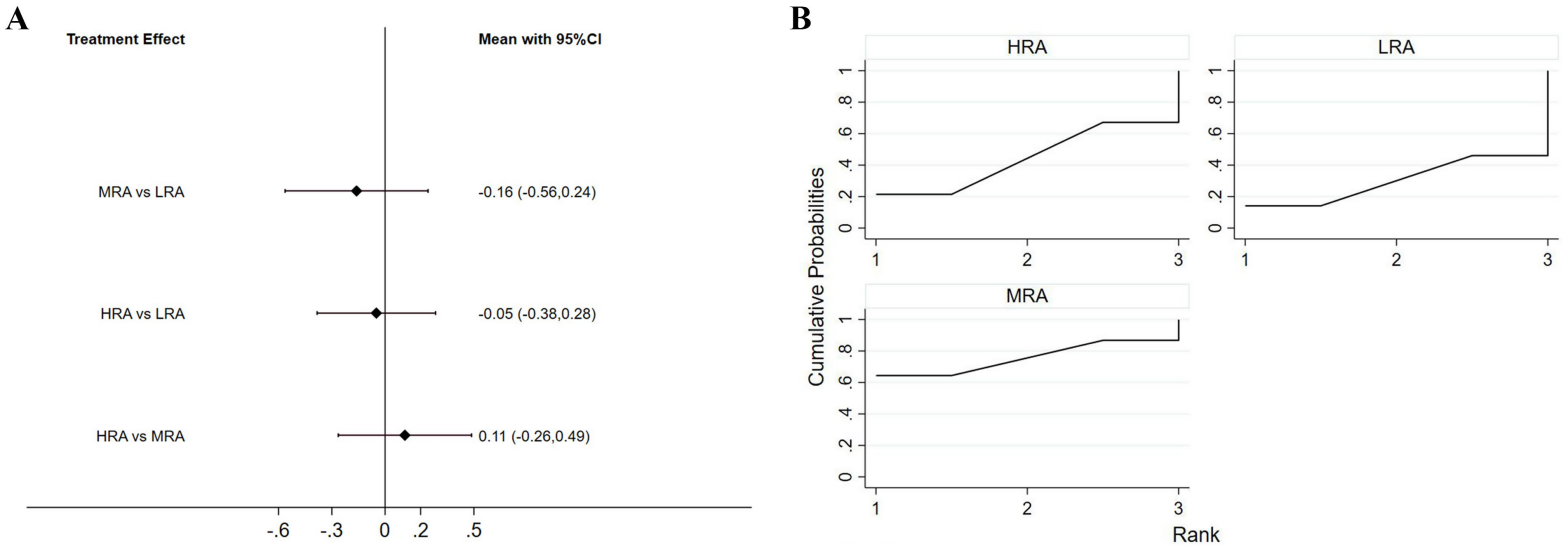
**(A)** Interval diagram based on eeriness. **(B)** Surface under the cumulative ranking curve based on eeriness.

Five studies (38.4%) included comparisons of eeriness. The NMA results showed that although the MRA (*p* = 0.433) and the HRA (*p* = 0.766) tended to be better than the LRA, these differences were not statistically significant at the *α* = 0.05 level. No significant difference was observed between the MRA and HRA either (Figure 5A).

The SUCRA rankings for each level of VAAR for eeriness showed that MRA had the highest probability of being perceived as the most eerie (74.5%), followed by HRA (44.7%) and LRA (30.9%) (Figure 5B).

### 3.8 Subgroup analysis of the effects of avatar presentation modes on interaction experience

Supplementary material 2 presents the cumulative ranking curves of different presentation media in examining the impact of VAAR on interaction experience. We conducted subgroup network meta-analyses separately for VR and non-VR conditions (e.g., video). The results under non-VR conditions showed that the SUCRA rankings for attractiveness and trustworthiness were HRA > LRA > MRA. For eeriness, the pattern was consistent with the main analysis, namely MRA > HRA > LRA.

Under VR conditions, the results were consistent with the main analysis: HRA were clearly superior to MRA and LRA in both attractiveness and trustworthiness, with the SUCRA ranking being HRA > MRA > LRA. For eeriness, however, the ranking was MRA > LRA > HRA. These findings suggest that the presentation medium may serve as a moderating factor in the relationship between VAAR and interaction experience.

### 3.9 Detection of publication bias

Funnel plots were generated for each outcome indicator to visually assess potential publication bias [Supplementary material 1(e)]. These plots appeared generally symmetrical, with data points evenly distributed within the inverted funnel shape, suggesting a low likelihood of small-study effects or publication bias.

## 4 Discussion

This study focuses on the impact of VAAR on user perceptual interaction experience, by systematically comparing subjective evaluations across different levels of VAAR. As shown in the SUCRA cumulative ranking plot, HRA received the highest ratings for both visual attractiveness and trustworthiness compared to other levels of VAAR, while MRA was associated with stronger perceptions of eeriness.

### 4.1 Visual attractiveness

In this study, we found that HRA received significantly higher ratings in visual attractiveness compared with MRA and LRA. This outcome may be attributed to the ability of HRA to reproduce body proportions and appearance details that closely resemble those of real humans. When VAAR provides highly realistic visual features that align with users’ perceptual expectations, these avatars can be recognized more quickly and easily. Such resemblance enhances users’ processing fluency during interactions with HRA, and it has been shown to elicit more positive affective responses as well as stronger judgments of attractiveness (Reber et al., 2004; Zibrek et al., 2018).

In contrast, the lower ratings of MRA and LRA may be attributed to the following reasons: MRA lacks sufficient visual detail, whereas LRA is overly exaggerated and stylized, with proportions and features that deviate considerably from human aesthetic standards. As a result, neither type of avatar fully aligns with users’ aesthetic and cognitive expectations. This not only weakens their initial attractiveness but also makes it difficult to sustain users’ interest and preference over long-term interactions.

### 4.2 Trustworthiness

Consistent with findings on visual attractiveness, users’ perceptions of avatar trustworthiness are significantly influenced by the level of VAAR. Our results indicate that HRA received higher trustworthiness ratings than MRA and LRA, which is similar to prior findings (Canales et al., 2024; Cornelius et al., 2023; Seymour et al., 2021). Prior research has demonstrated that users are more inclined to engage with virtual avatars that appear realistic and credible (Yoon et al., 2019). In domains that emphasize professionalism and authority, HRAs can enhance credibility not only through anthropomorphic features but also by integrating context-appropriate visual cues (e.g., a doctor’s white coat) and environmental settings (e.g., a hospital background), thereby reinforcing users’ sense of trustworthiness and social identity.

We argue that this enhancement of trustworthiness at the social and contextual level can be largely explained from a neural mechanism perspective. Prior research has shown that the fusiform face area (FFA) and the prefrontal cortex (PFC) are closely involved in the neural processes underlying trust (Delgado et al., 2005; Kanwisher et al., 1997). The FFA, located in the occipitotemporal cortex, is primarily responsible for the rapid recognition and categorization of faces, enabling individuals to distinguish between familiar and unfamiliar, as well as trustworthy and untrustworthy (Haxby et al., 2000; Kanwisher et al., 1997). The PFC integrates inputs from the FFA with contextual, emotional, mnemonic, and social information, playing a critical role in social evaluation and trust judgments (Amodio and Frith, 2006). Therefore, we suggest that the high VAAR and contextually appropriate visual features of HRAs make them closely resemble real social agents, facilitating rapid processing by the FFA. Once processed facial signals are transmitted to the PFC for social evaluation, they are more readily assigned to the designated social role category, thereby strengthening their perceived trustworthiness. In contrast, MRAs and LRAs, due to their limited detail or overly abstract design, are less likely to establish efficient processing pathways between the FFA and PFC. Consequently, they are less capable of eliciting strong trust responses.

In summary, HRAs combined with congruent visual cues are more likely to enhance users’ perceptions of trustworthiness. It is noteworthy that as artificial intelligence (AI) is increasingly embedded in contexts such as medical consultation and educational training, the visual professionalism of AI avatars has become a critical factor influencing whether users adopt the information they provide. Unlike traditional human interactions, AI systems often lack the social cues and reputation support that humans rely on. Therefore, users tend to place greater emphasis on appearance as an initial cue for evaluating trustworthiness. By incorporating high realism with contextually appropriate visual elements, HRAs can help users quickly establish trust in the absence of familiarity, thereby increasing the persuasiveness and acceptance of AI systems in professional contexts.

### 4.3 Eeriness

**Figure 6:**
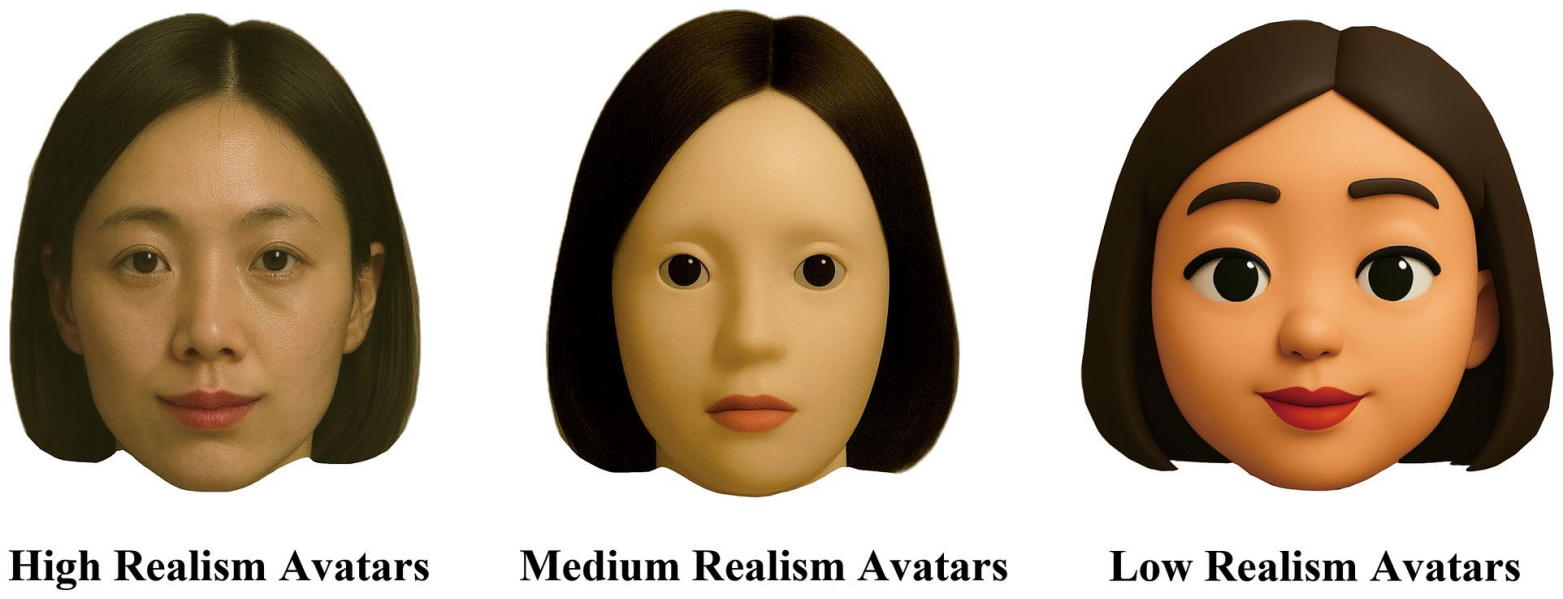
Illustration of virtual avatars with low, medium, and high levels of realism.

Unlike the findings on visual attractiveness and trustworthiness, the relationship between VAAR and perceived eeriness did not exhibit a linear positive trend. Our results showed that MRA received higher eeriness ratings compared to HRA and LRA, which suggests that they are most likely to induce feelings of unease. This finding is consistent with the explanation of the “uncanny valley” effect: although MRAs possess many human-like external features, the lack of natural coherence at the detail level makes them more prone to eliciting discomfort (MacDorman and Ishiguro, 2006; Mori et al., 2012). As illustrated in Figure 6, the misaligned features of MRA are particularly prominent among the three avatar realism levels, such as abnormal eye details, overly smooth skin texture, and rigid facial expressions.

Moreover, the experience of eeriness does not stem solely from visual incongruence. Even highly realistic HRA may evoke discomfort if they lack coordination and integration across visual and behavioral dimensions (Dubosc et al., 2023; MacDorman et al., 2009; Zibrek et al., 2018). This phenomenon can be explained by the avoidance mechanisms developed during human evolution. Such mechanisms enable the rapid visual detection of potential threats, whereby abnormal appearances and atypical behaviors are perceived as warning signals that trigger instinctive vigilance and avoidance (Curtis et al., 2004). Accordingly, when individuals encounter MRA, abnormalities in appearance or behavioral details may be classified by the brain as potential threats, thereby eliciting feelings of disgust and rejection.

In summary, the formation of eeriness is not merely an aesthetic response to appearance but is influenced by multiple complex psychological mechanisms. In future efforts to enhance the realism of virtual avatars, designers should not only focus on visual fidelity in appearance but also emphasize naturalness in movements, expressions, and cross-modal consistency, in order to more effectively mitigate the uncanny valley effect associated with avatars.

### 4.4 Subgroup analysis

It is noteworthy that the presentation medium itself may constitute an important source of heterogeneity. Subgroup analyses revealed that under non-VR conditions, LRA ranked higher than expected, particularly in the dimensions of attractiveness and trustworthiness, even surpassing MRA, whereas in VR conditions, the ranking pattern followed HRA > MRA > LRA. This discrepancy may be related to users’ long-term media habits: people are accustomed to encountering low-realism characters such as anime or cartoons through flat media like smartphones and television, and this accumulated experience makes LRA easier to accept and interpret, sometimes even associated with positive aesthetic connotations (Matsuda et al., 2015). In contrast, in VR environments, the heightened sense of presence and interactive cues not only strengthen the advantage of HRA but also partially alleviate the discomfort of MRA. However, the overly simplified and cartoon-like features of LRA may clash with the highly realistic three-dimensional virtual environment, thereby diminishing attractiveness and trustworthiness. Therefore, the presentation medium plays a critical role in moderating the effects of VAAR on user experience.

### 4.5 Implications for virtual avatars and artificial intelligence

AI-driven virtual avatar assistants have become increasingly integrated into people’s daily lives, with applications spanning medical consultation, educational training, financial services, and entertainment. For example, Doubao, launched by China’s Douyin, and AI companions such as Replika have attracted widespread attention globally. Our findings indicate that the visual realism of virtual avatars not only shapes users’ perceptions of attractiveness but also directly influences their sense of trust. In contrast, MRA often induce feelings of uncanniness due to insufficient detail or disproportionate features, which diminish interaction experiences and should therefore be avoided. Moreover, the mode of presentation (e.g., video versus VR) can also affect users’ interactions with avatars to some extent. Therefore, AI systems should adapt avatar design according to the application context: emphasizing realism and professionalism in high-risk or expertise-driven scenarios, while employing stylized and personalized features in entertainment and creative contexts, to enhance trustworthiness and attractiveness while mitigating the “uncanny valley” effect.

### 4.6 Limitations

Although the present NMA synthesized current evidence on the relationship between VAAR and users’ perceptual interaction experiences, several limitations should be acknowledged before drawing firm conclusions. First, this study primarily relied on subjective self-reported evaluations and lacked objective indicators derived from physiological signals. Second, although the overall sample size was relatively large, some core studies included small sample sizes and demonstrated limited demographic representativeness, particularly with respect to female and non-Western participants. Furthermore, while this study incorporated research from China, Netherlands, Australia, France, Ireland, Germany, and the United States, the overall geographic and cultural diversity remained insufficient, with evidence from Africa, Latin America, and the Middle East almost entirely absent. Such limitations may raise concerns about the generalizability of the findings. Finally, this review only included publications in English, which may have introduced publication bias and restricted the applicability of the results to broader populations.

### 4.7 Future research

Future research should further adopt multimodal measurement approaches by combining subjective self-reports with physiological indicators to more comprehensively capture users’ perception of avatar realism and to validate the reliability of subjective evaluations. At the same time, greater demographic and cultural diversity is needed in sample composition, not only ensuring gender balance but also expanding participant recruitment to other regions to enhance the cross-cultural generalizability of the findings. In addition, strengthening multilingual and cross-regional collaboration would help reduce linguistic and geographic biases. Meanwhile, systematic investigations of AI-driven virtual assistants or companion avatars remain scarce. Future studies should further examine the unique mechanisms of AI avatars across various domains to uncover how their realism influences user experience.

## 5 Conclusion

Virtual avatars with high VAAR are generally perceived as more attractive and trustworthy. In contrast, avatars with medium VAAR are most likely to elicit a sense of eeriness, potentially resulting in negative user experiences. Moreover, the virtual avatars presentation mode can also affect users’ interactions with it to some extent. This study offers valuable guidance for optimizing the design of virtual avatars.

## Data Availability

The original contributions presented in the study are included in the article/Supplementary material, further inquiries can be directed to the corresponding author.
