# Supplementary material for: Impact of virtual avatar appearance realism on perceptual interaction experience: a network meta-analysis
Source: Front Psychol. 2025 Dec 3;16:1624975. doi: 10.3389/fpsyg.2025.1624975 (PMC12709275; doi:10.3389/fpsyg.2025.1624975)

(a) Search strategy

**Web of Science**

#1 TS= ("Virtual Human" OR "Digital Human" OR "Virtual Character" OR "Virtual Avatars")

#2 TS= ("Appearance Realism" OR "Visual Realism" OR "Photorealism" OR "Facial Realism")

#3 #1 AND #2

#4 #3 AND Publication Year= (2025 OR 2024 OR 2023 OR 2022 OR 2021 OR 2020 OR 2019 OR 2018 OR 2017 OR 2016 OR 2015 )

**ScienceDirect**

#1 ("Virtual Human" OR "Digital Human" OR "Virtual Character" OR "Virtual Avatars") [in Title, Abstract, Keywords]

#2 ("Appearance Realism" OR "Visual Realism" OR "Photorealism" OR "Facial Realism") [in Title, Abstract, Keywords]

#3 #1 AND #2

#4 Filters: Year: 2015–2025, Article type: Research Articles

**SpringerLink**

#1 ("Virtual Human" OR "Digital Human" OR "Virtual Character" OR "Virtual Avatars")

#2 ("Appearance Realism" OR "Visual Realism" OR "Photorealism" OR "Facial Realism")

#3 #1 AND #2

#4 Filters applied: Article type = Journal Article, Year = 2015–2025

**Google Scholar**

#1 All in one query:

("Virtual Human" OR "Digital Human" OR "Virtual Character" OR "Virtual Avatars") AND

("Appearance Realism" OR "Visual Realism" OR "Photorealism" OR "Facial Realism")

#2 Custom date range: From 2015 to 2025

#3 Sorted by relevance; no additional filters applie

| Criterion | Rating Description (1–5 points) |
| --- | --- |
| Skin Texture | 1 = Completely flat, uniform color, cartoon-like rendering; 2 = Basic shading, lacking details; 3 = Rough or blurred texture; 4 = Noticeable pores, gradual skin tone changes; 5 = High resolution, detailed pores, wrinkles, and natural highlights/shadows |
| Facial Proportions | 1 = Extremely exaggerated or cartoon-like; 2 = Somewhat distorted but still recognizable as a “face”; 3 = Generally proportional but stylized; 4 = Basically conforms to realistic human facial proportions; 5 = Highly realistic, consistent with an actual human face |
| Body Proportions | 1 = Distorted or deformed; 2 = Basic humanoid but with unrealistic proportions; 3 = Generally correct but stylized; 4 = Close to realistic human proportions; 5 = Fully conforms to anatomical proportions |
| Stylization Level | 1 = Clearly cartoonish, low-poly, comic or anime style; 2 = Highly simplified, lacking realistic details; 3 = A mix of realism and cartoon stylization; 4 = Close to realism, only slight stylization; 5 = Fully realistic |

(b) VAAR evaluates scale

| Author | EC1 | EC2 | EC3 | EC4 | EC5 | EC6 | EC7 | EC8 | EC9 | EC10 | Total score |
| --- | --- | --- | --- | --- | --- | --- | --- | --- | --- | --- | --- |
| Dubosc et al. | 1 | 1 | 1 | 1 | 1 | 0 | 1 | 1 | 1 | 1 | 9 |
| Seymour et al. | 1 | 1 | 1 | 1 | 1 | 0 | 1 | 1 | 1 | 1 | 9 |
| Cornelius et al. | 1 | 1 | 1 | 0 | 1 | 0 | 1 | 1 | 1 | 1 | 8 |
| Kokkinara et al. | 1 | 1 | 1 | 1 | 1 | 0 | 1 | 1 | 1 | 1 | 9 |
| Gorisse et al. | 1 | 1 | 1 | 1 | 1 | 1 | 1 | 1 | 1 | 1 | 10 |
| Amadou et al. | 1 | 1 | 1 | 1 | 1 | 1 | 1 | 1 | 1 | 1 | 10 |
| Mal et al. | 1 | 1 | 1 | 1 | 1 | 0 | 1 | 1 | 1 | 1 | 9 |
| Stein et al. | 1 | 1 | 1 | 1 | 1 | 0 | 1 | 1 | 1 | 1 | 9 |
| Straßmann et al. | 1 | 1 | 1 | 1 | 1 | 1 | 1 | 1 | 1 | 1 | 10 |
| Zibrek et al. | 1 | 1 | 1 | 1 | 1 | 1 | 1 | 1 | 1 | 1 | 10 |
| Canales et al. | 1 | 1 | 1 | 0 | 1 | 1 | 1 | 1 | 1 | 1 | 9 |
| Gao et al. | 1 | 1 | 1 | 1 | 1 | 0 | 1 | 1 | 1 | 1 | 9 |
| Fraser et al. | 1 | 1 | 1 | 1 | 1 | 0 | 1 | 1 | 1 | 1 | 9 |

(c) Methodological quality assessment items and corresponding results

| The Evaluation Criteria (EC) presented below: |
| --- |
| EC1: The aim of the study was clearly defined. |
| EC2: The context in which the study was conducted adequately described. |
| EC3: The research design was appropriate for addressing the study aims. |
| EC4: The characteristics of participants were clearly defined. |
| EC5: The data collection methods of the study were thoroughly described. |
| EC6: The study has received at least 10 citations. |
| EC7: Study provided a detailed description and justification of the data analysis procedures. |
| EC8: Results of the study were clearly presented. |
| EC9: Discussion and conclusion clearly compare the findings of the study with existing literature. |
| EC10: The study contributes to existing literature.  (d) Basic characteristics of each study |

(e) Publication bias


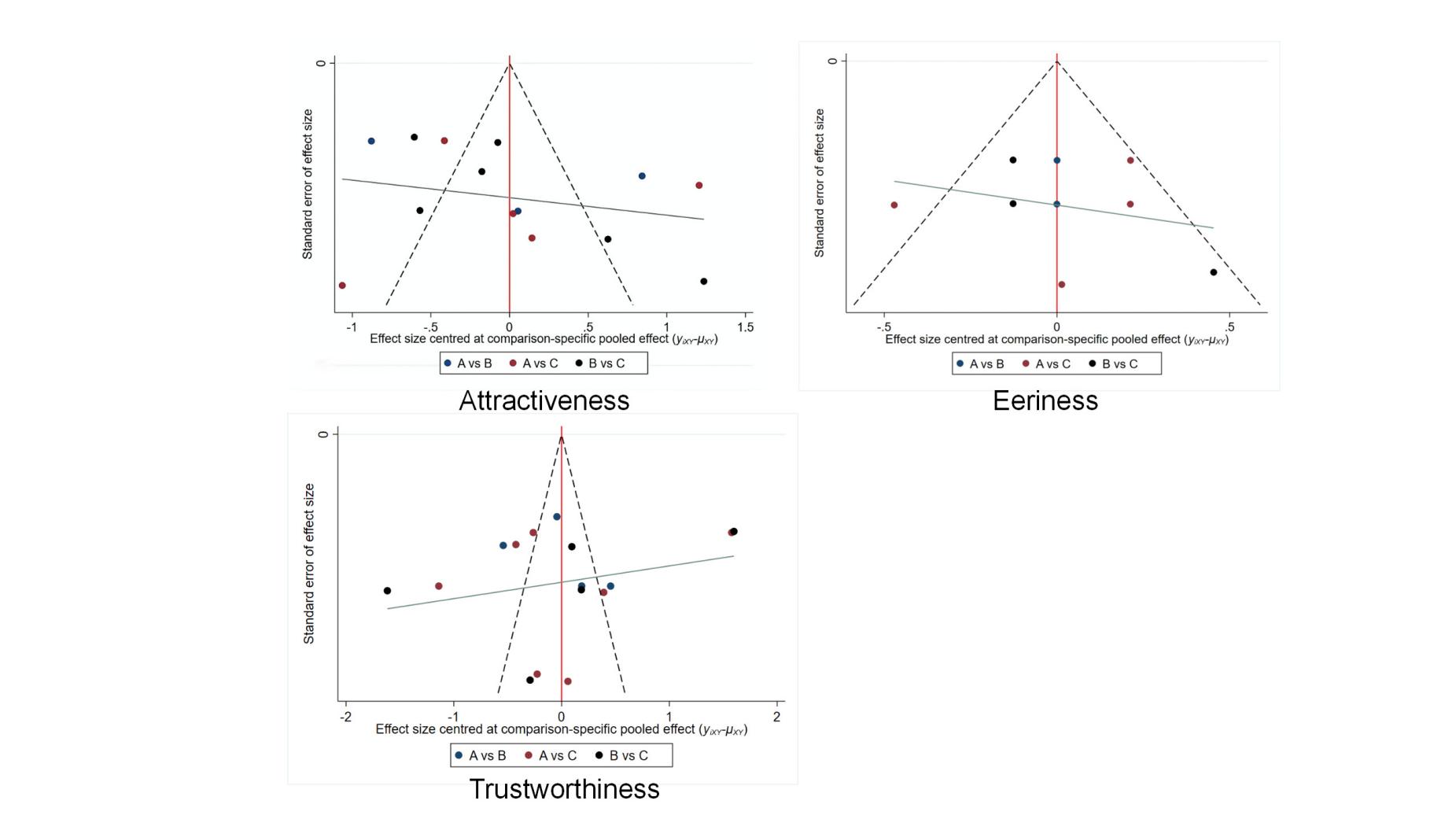

Supplement: Supplementary file 1 [file Data_Sheet_1.DOCX]
