# Supplementary material for: Impact of virtual avatar appearance realism on perceptual interaction experience: a network meta-analysis
Source: Front Psychol. 2025 Dec 3;16:1624975. doi: 10.3389/fpsyg.2025.1624975 (PMC12709275; doi:10.3389/fpsyg.2025.1624975)
Supplement: Supplementary file 2 [file Data_Sheet_2.DOCX]

**Attractiveness**

Non-VR presentation Ranking


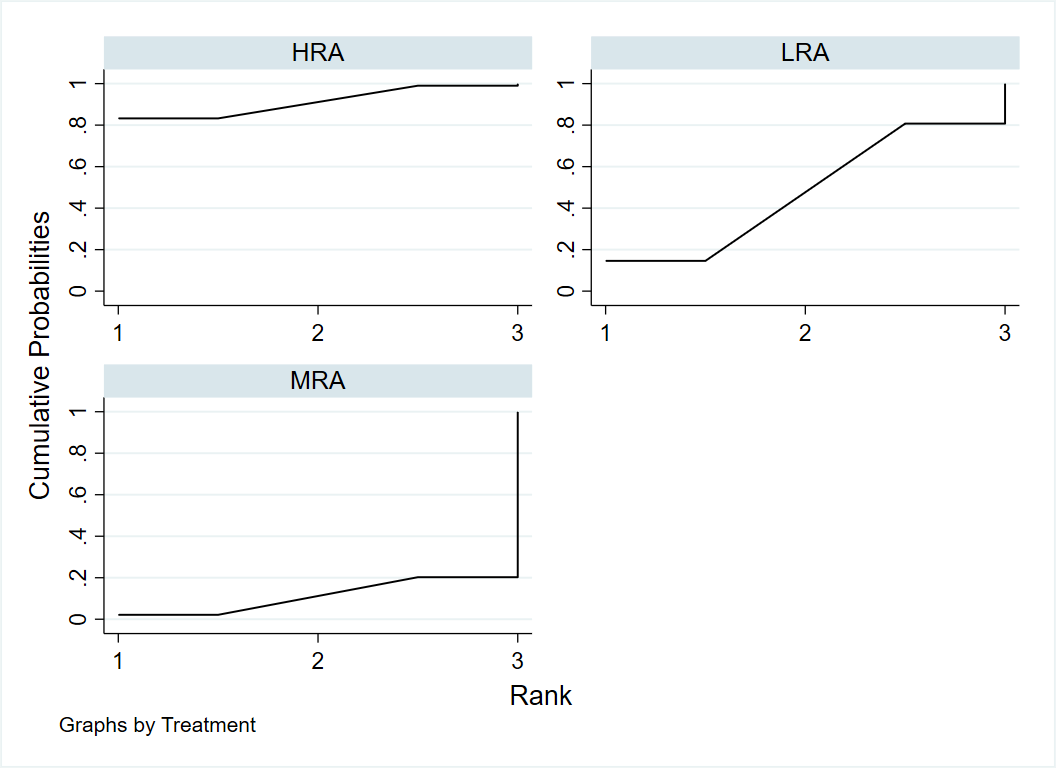


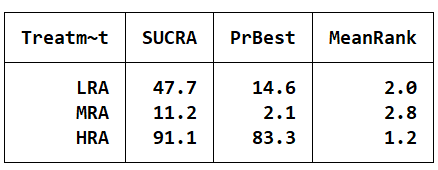


VR presentation Ranking


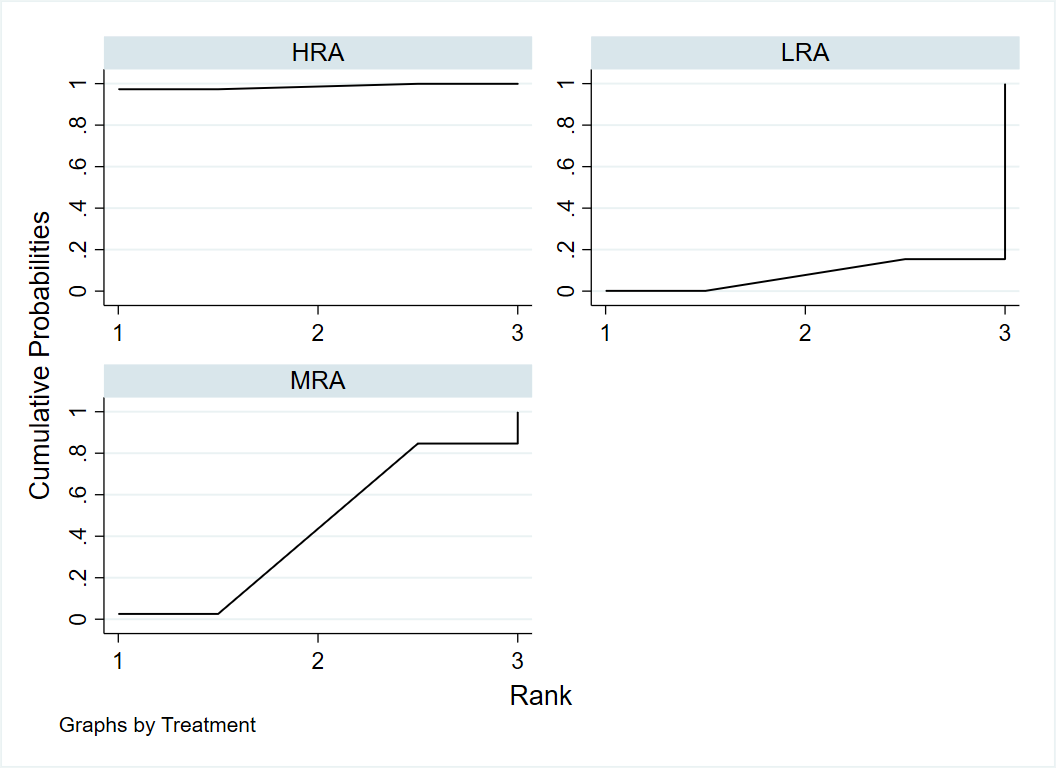


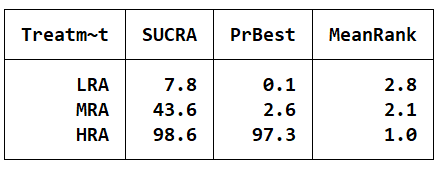


**Trustworthiness**

Non-VR presentation Ranking


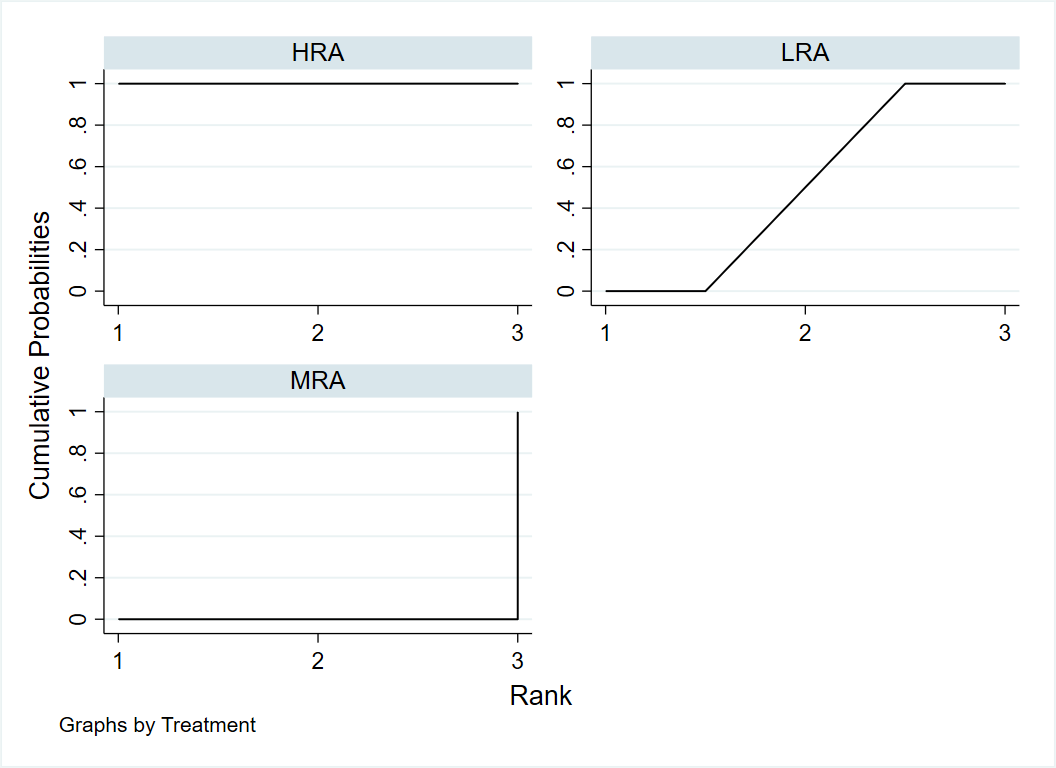


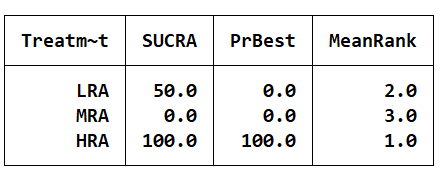


VR presentation Ranking


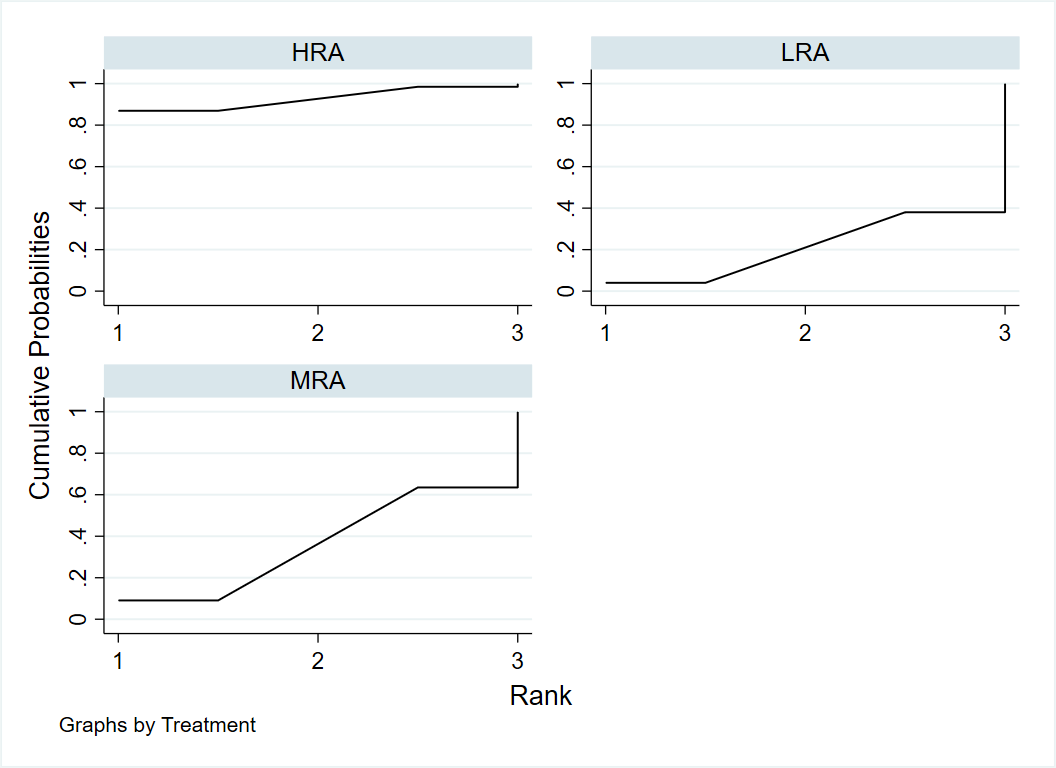


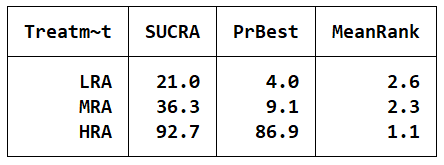


**Eeriness**

Non-VR presentation Ranking


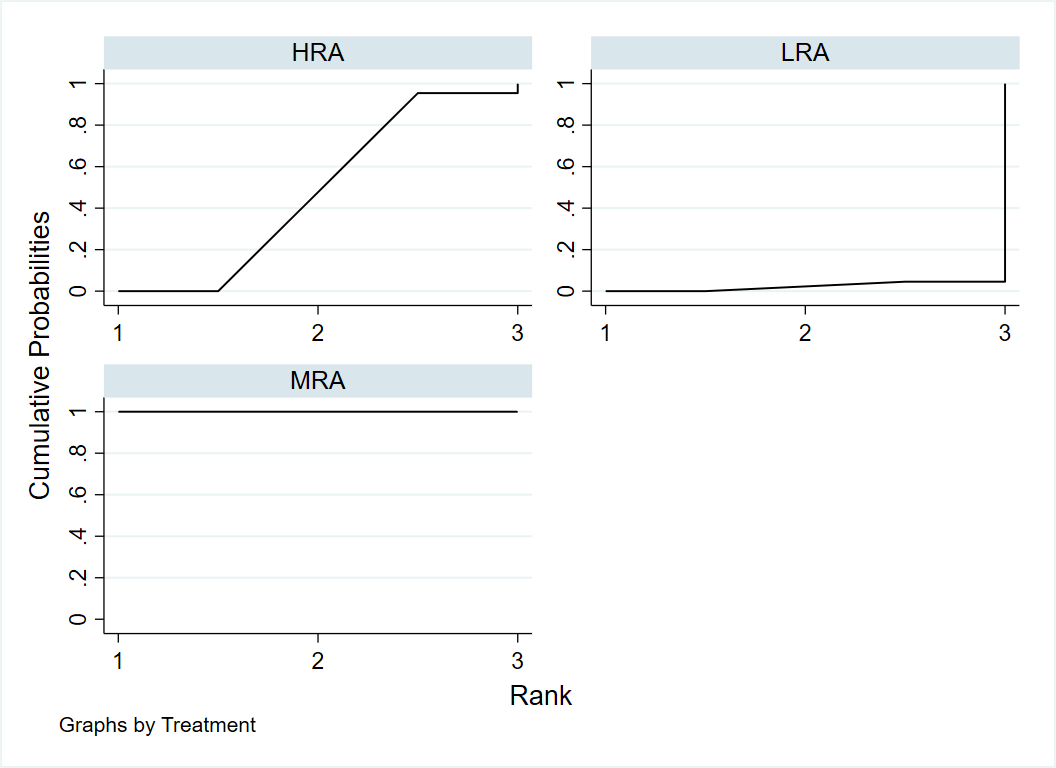


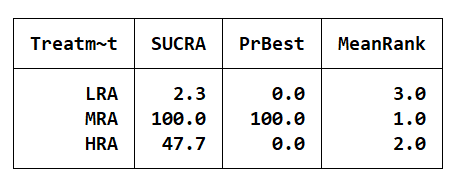


VR presentation Ranking

**
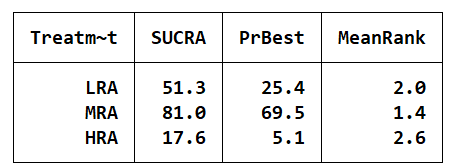

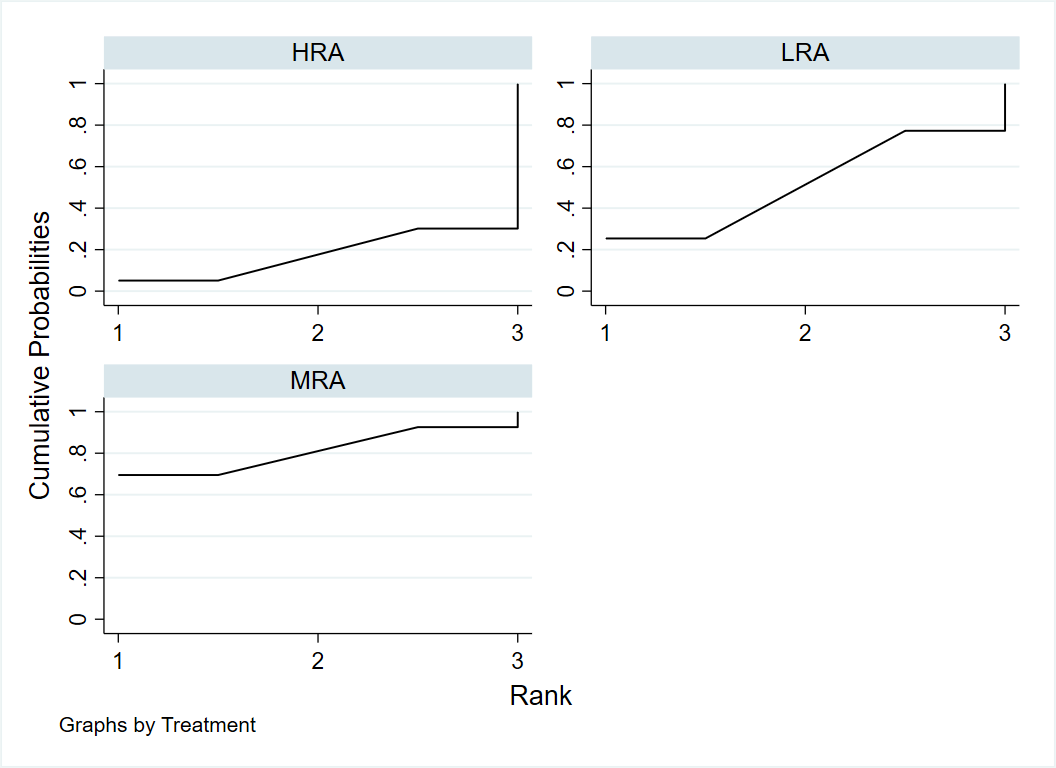
**
